# Establishment and validation of a radiological-radiomics model for predicting high-grade patterns of lung adenocarcinoma less than or equal to 3 cm

**DOI:** 10.3389/fonc.2022.964322

**Published:** 2022-09-15

**Authors:** Hao Dong, Lekang Yin, Lei Chen, Qingle Wang, Xianpan Pan, Yang Li, Xiaodan Ye, Mengsu Zeng

**Affiliations:** ^1^ Department of Radiology, First People’s Hospital of Xiaoshan District, Hangzhou, China; ^2^ Department of Radiology, Zhongshan Hospital, Fudan University, Shanghai, China; ^3^ Department of Research, Shanghai United Imaging Intelligence Co. Ltd., Shanghai, China; ^4^ Shanghai Institute of Medical Imaging, Shanghai, China; ^5^ Department of Cancer Center, Zhongshan Hospital, Fudan University, Shanghai, China

**Keywords:** lung adenocarcinoma, high-grade pattern, radiomics, model, predictive performance

## Abstract

**Objective:**

We aimed to develop a Radiological-Radiomics (R-R) based model for predicting the high-grade pattern (HGP) of lung adenocarcinoma and evaluate its predictive performance.

**Methods:**

The clinical, pathological, and imaging data of 374 patients pathologically confirmed with lung adenocarcinoma (374 lesions in total) were retrospectively analyzed. The 374 lesions were assigned to HGP (n = 81) and non-high-grade pattern (n-HGP, n = 293) groups depending on the presence or absence of high-grade components in pathological findings. The least absolute shrinkage and selection operator (LASSO) method was utilized to screen features on the United Imaging artificial intelligence scientific research platform, and logistic regression models for predicting HGP were constructed, namely, Radiological model, Radiomics model, and R-R model. Also, receiver operating curve (ROC) curves were plotted on the platform, generating corresponding area under the curve (AUC), sensitivity, specificity, and accuracy. Using the platform, nomograms for R-R models were also provided, and calibration curves and decision curves were drawn to evaluate the performance and clinical utility of the model. The statistical differences in the performance of the models were compared by the DeLong test.

**Results:**

The R-R model for HGP prediction achieved an AUC value of 0.923 (95% CI: 0.891-0.948), a sensitivity of 87.0%, a specificity of 83.4%, and an accuracy of 84.2% in the training set. In the validation set, this model exhibited an AUC value of 0.920 (95% CI: 0.887-0.945), a sensitivity of 87.5%, a specificity of 83.3%, and an accuracy of 84.2%. The DeLong test demonstrated optimal performance of the R-R model among the three models, and decision curves validated the clinical utility of the R-R model.

**Conclusion:**

In this study, we developed a fusion model using radiomic features combined with radiological features to predict the high-grade pattern of lung adenocarcinoma, and this model shows excellent diagnostic performance. The R-R model can provide certain guidance for clinical diagnosis and surgical treatment plans, contributing to improving the prognosis of patients.

## Introduction

Lung cancer represents the leading cause of cancer-associated morbidity and mortality worldwide (1). Non-small cell lung cancer (NSCLC) constitutes over 80% of lung cancers, of which lung adenocarcinoma (LUAD) is known as the most common histological subtype (2). According to the 2015 World Health Organization Classification, LUADs are categorized into three prognostic subsets on the ground of the predominant histological pattern: low-grade (lepidic-predominantly), intermediate-grade (acinar- or papillary-predominant), and high-grade (solid or micropapillary-predominant) (3). However, prior studies have indicated that the actual prognosis varies largely even among LUADs presenting the same predominant pattern (4–6). The latest study has reported that even among early-stage LUAD patients, patients with a minimal high-grade pattern (HGP) (micropapillary or solid) have poorer outcomes (7).

Generally, radical resection has been the optimal treatment for LUAD (8). However, the presence of micropapillary or solid component (HGP) is considered an independent predictor of postoperative local recurrence (9, 10). LUAD patients with an HGP may require extensive surgical resection and more aggressive adjuvant chemotherapy (11–13). Although an HGP correlates with a worse prognosis, it is difficult to identify the presence of HGP prior to surgery. Owing to the extensive heterogeneity of LUAD, LUAD subtypes may be underestimated by either preoperative puncture pathology or intraoperative frozen pathology limited by sampling (14). In addition, there are rare cases presenting a predominant HGP in our clinical practice; instead, more cases have a pathology containing an HGP, which leads to greater difficulty in pathological examination of the high-grade components. Therefore, determining the presence of any HGPs in LUAD both preoperatively and intraoperatively is a clinically meaningful and challenging task.

To predict the presence of a high-grade patterns in lung adenocarcinoma, Choi’s study (15) showed that the CT values of the tumors were meaningful and lower HU (Hounsfield) values were associated with a lower-grade histological pattern (OR = 6.15, p = 0.005). Additionally, SUVmax of the tumor was associated with high-grade patterns (OR = 1.14, p = 0.012). However, our study used the radiomics method for further accurate prediction of high-grade patterns. Radiomics analysis is a fast-rising powerful tool in the field of medical image analysis in recent years. It is a robust and objective method that quantifies the high-dimensional tumor features undetectable with the naked eye, as compared to subjective imaging evaluation (16, 17). It is also a non-invasive, quantitative method for tumor heterogeneity assessment that can be applied to quantify intratumoral heterogeneity. Recent studies have employed radiomics-based methods to predict high-grade components of LUAD (18–20), whereas, the present study used a larger sample size and added traditional radiological features to develop a model for comparison, and combined with radiomics features, constructed an integrated model to predict the HGP of LUAD. In this study, we aimed to develop and evaluate the value of a radiological-radiomics model for predicting the HGP in LUAD less than or equal to 3 cm.

## Materials and methods

### Clinical data

**Figure 1:**
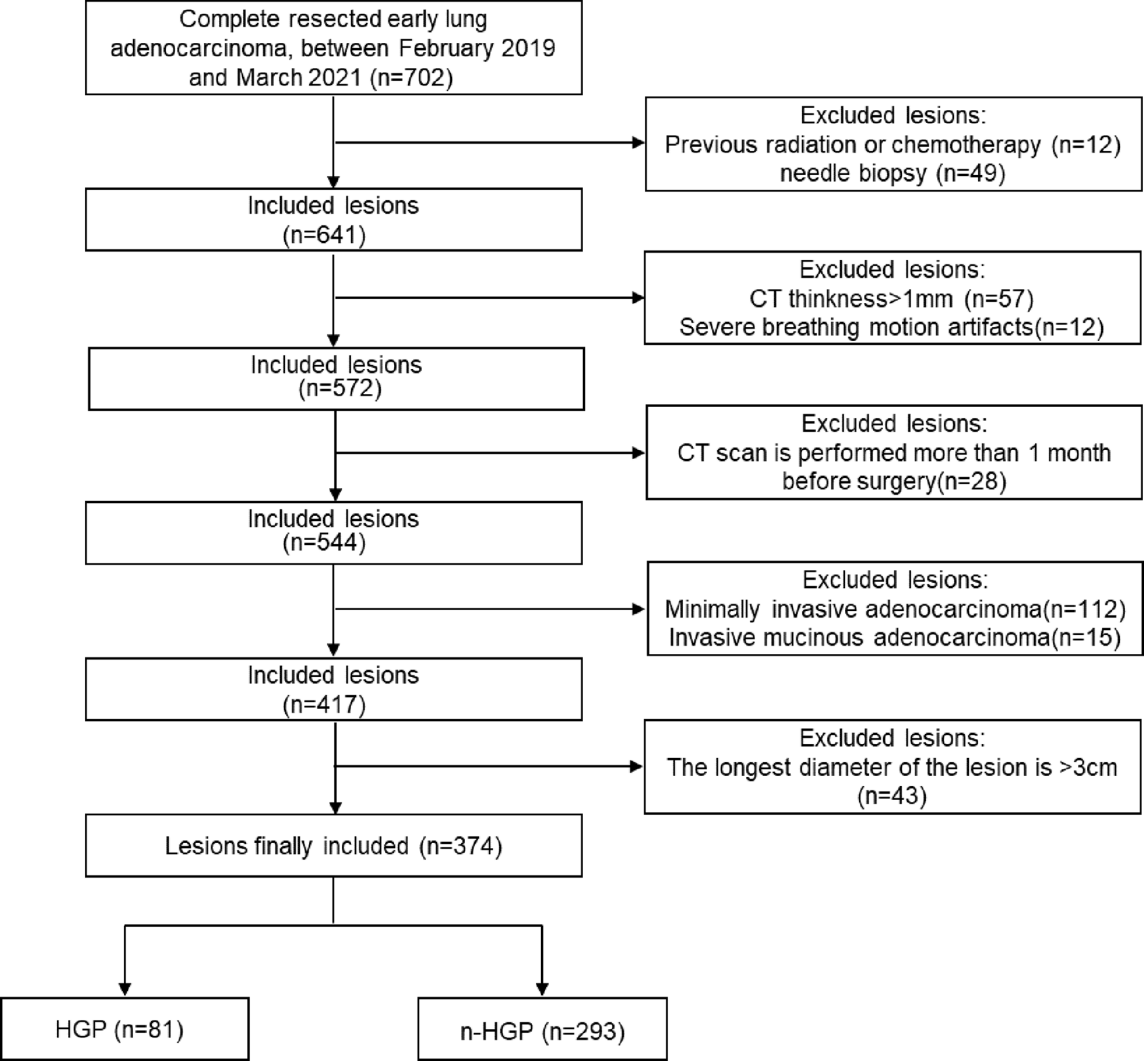
Schedule of patient enrolment.

The clinical, pathological, and CT data of patients with LUAD who underwent surgical resection in the department of thoracic surgery, Zhongshan Hospital Affiliated to Fudan University from February 2019 to March 2021 were retrospectively analyzed. Inclusion criteria: 1) patients did not receive any chemoradiotherapy or needle biopsy before computed tomography (CT) examination; 2) patients had high-resolution computed tomography (HRCT) examination images (slice thickness ≤ 1 mm) within 1 month before surgery. Exclusion criteria: 1) severe respiratory motion artifacts; 2) the longest diameter of the lesions > 3 cm (3); minimally invasive adenocarcinoma and invasive mucinous adenocarcinoma. Ultimately, 374 lesions from 374 patients (123 males and 251 females) were enrolled. The patients were aged 25-87 with a mean age of (56 ± 11) years. When multiple lesions were present in the patient’s postoperative specimens, only the main lesion (the one with the largest diameter) was chosen for this experiment. The patient enrollment flowchart is described in **Figure 1**. The clinical and pathological data of patients contained age, gender, smoking history, tumor location, tumor stage, presence/absence of lymph node or pleural metastasis, and presence/absence of tumor spread through air spaces (STAS), etc. Tumor node metastasis (TNM) staging was based on the IASLC TNM staging system for lung cancer, 8^th^ edition (21). The 374 lesions were sub-grouped into HGP (HGP) and non-HGP (n-HGP) groups in the presence or absence of HGP in pathological findings. This study was approved by the Ethics Committee of Zhongshan Hospital Affiliated to Fudan University.

### Examination methods

The Siemens SOMATOM Definition AS+128-slice spiral CT scanner and Philips Brilliance 64-slice spiral CT scanner were applied for scanning. Scanning parameters of the Siemens CT scanner were set as follows: tube voltage 120 KV, tube current 250 mA, pitch 1.1, reconstruction slice thickness 1 mm, reconstruction interval 1 mm. Philips CT scanner parameters: tube voltage 130 KV, tube current 200 mA, pitch 0.64, reconstruction slice thickness 1mm, reconstruction interval 1 mm. All patients underwent breath-holding training before scanning. During the scanning, breath-holding was required under free-breathing. The scan range was from the apex of the lung to the base of the lung, covering the axilla and chest wall on both sides. The bone algorithm was employed for reconstruction.

### Radiological analysis

CT images were independently retrospectively analyzed by two radiologists with 8 years and 25 years of chest CT experience, blinded to the pathological results. A consensus will be reached after a discussion on disagreement. Based on CT images, the lesions were described in terms of lesion location, size, density, shape, spiculation, lobulation, vacuole, air bronchogram, and pleural indentation. The definitions of specific CT features are outlined in **Table 1**.

**Table 1:** CT features for lung adenocarcinoma.

Variable	Definition
Density	SN(solid nodule): Circular or quasi-circular increased density shadows in the lungs, the lesions are dense enough to cover the blood vessels and bronchial shadows running in them; SSN(subsolid nodule):All pulmonary nodules with ground-glass density are called SSN. Ground-glass lesions refer to CT with clear or indistinct borders, but the density of the lesions is not enough to cover the blood vessels and bronchi
Shape	Indicated as lobulated, others (round, or oval)
Lobulation	The surface of the tumor showed as multiple arc-shaped projections
Spiculation	Evaluated in the lung window, and indicated as different degrees of spinous or burr-like protrusions at the tumor margin
Vacuole	Single or multiple small punctate hypodense shadows less than 5mm in the tumor
Air bronchogram	Tube like or branched air structure within the tumor
Pleural indentation	Retraction of the pleura towards the tumor

### Radiomics analysis

Lesion segmentation: lung window image sequences of patients were imported into the uAI artificial intelligence scientific research platform (uAI Research Portal; United Imaging Intelligence, Shanghai, China) in DICOM format. Initially, preprocessing such as anonymization and image normalization was performed, followed by automatic detection and segmentation of lung nodule volume of interest (VOI) with the platform built-in models. This model is a VB-Net deep learning model based on its own intellectual property rights. Using an integrated two-level network based on images and feature pyramid networks (FPN), this model has been trained and tested on multi-center datasets (its intelligent auxiliary detection software for lung nodules taking this model as the core has attained the NMPA Class III certificate). The segmentation results of pulmonary nodules were output with the platform built-in deep learning model and jointly confirmed layer by layer by two radiologists with 8 years and 15 years of lung cancer imaging diagnosis experience for necessary modifications (radiologists did not refer to the pathological results). The VOI was delineated depending on the tumor-lung interface, and the structures such as blood vessels and bronchi were excluded as much as possible during the delineation process.

Radiomics feature extraction: the radiomics features of tumor tissue within the VOI were calculated using the feature extraction function of the platform, in which the PyRadiomics toolkit (https://pyradiomics.readthedocs.io/en/latest/index.html) was embedded. The images were resampled with the pixel spacing of the images in the three anatomical directions as 1.0 mm, to eliminate the interference caused by the spatial resolution inconsistency attributable to different CT models. The original CT images were preprocessed with high-pass or low-pass wavelet filters and Laplacian Gaussian filters with different λ parameters, generating 8 wavelet-based preprocessed images and 5 Laplacian filters-based preprocessed images. The radiomic features of the original CT images and the preprocessed images were extracted, including first-order features based on CT values or pixel values of the preprocessed images, morphological features describing tumor morphology, and gray-level co-occurrence matrices (GLCM) describing tumor interior and surface texture, gray-level run-length matrix (GLRLM), gray-level size zone matrix (GLSZM), and gray-level difference matrix (GLDM) texture features. Ultimately, 2600 radiomics features were extracted for each lesion and normalized by Z-score.

**Figure 2:**
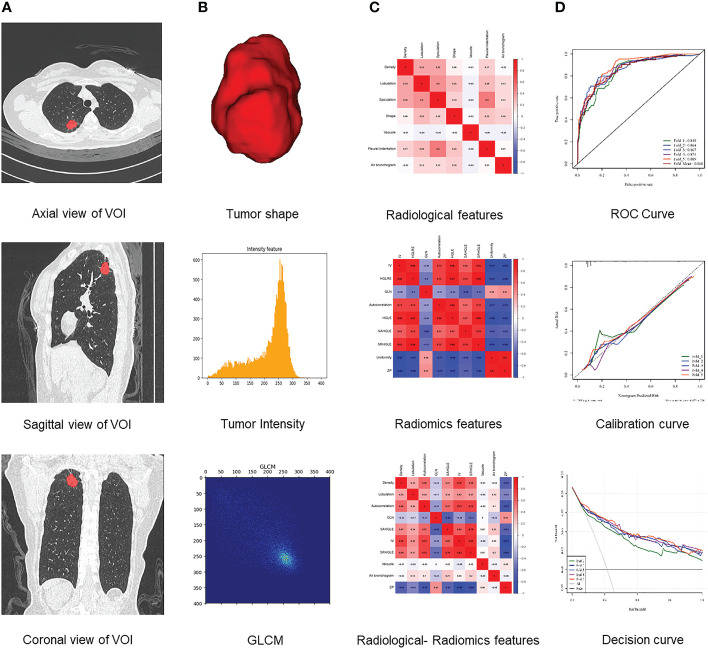
Flowchart of radiomics analysis. **(A)** Platform built-in lung nodule detection and segmentation model for automatic annotation of lung nodule VOI. **(B)** Features extracted from VOI, including tumor shape, intensity, and texture features. **(C)** Analysis of radiological, radiomics, and radiological-radiomics features. **(D)** Model establishment and evaluation.

Establishment and evaluation of a machine learning model: After the feature extraction, the least absolute shrinkage and selection operator (LASSO) method was applied for feature dimension reduction. Accordingly, the image feature dimension was reduced to less than 10% of the training data volume. Then, the machine learning classifier was adopted to build the models: the Radiological model was built using 7 radiological features, the Radiomics model was developed using the radiomics features selected by LASSO. Finally, the radiological features and the radiomics features were combined, and an R-R model was constructed. Multi-variable logistic regression method was selected as the classifier of the model, and the parameters of which were optimized using the grid-search method. Optimal parameters were reversely selected according to the area under the receiver operating curve (ROC) in the validation set. For each model, 5-fold cross-validation was employed for training and validation. We recorded the probability that the model could predict the presence of HGP in the training and validation set data at each iteration and calculated the mean values of the probabilities recorded at each iteration when each datum served as the training set or validation set, which were selected as the results of the corresponding set. Based on this, multiple indicators of the model in the training set and validation set were calculated, encompassing the area under the curve (AUC), accuracy, sensitivity, and specificity. Lastly, ROC, calibration, and decision curves were drawn for model evaluation. The aforesaid feature selection, model construction and evaluation were all carried out on the United Imaging scientific research platform. The flowchart of radiomics analysis is presented in **Figure 2**.

### Statistical analysis

The data were processed and statistically analyzed with SPSS 23.0 software (IBM, Armonk, NY, USA) and the United Imaging artificial intelligence scientific research platform (Shanghai United Imaging Healthcare Co., Ltd., Shanghai, China). Enumeration data between the two groups were compared by χ2 test or Fisher’s exact test. Logistic regression models (Scikit-learn software package) were constructed on the United Imaging artificial intelligence scientific research platform, designated as radiological model, radiomics model, and R-R model. ROC curves were plotted on this platform (Matplotlib software package), and the area under the curve (AUC), sensitivity, specificity, and accuracy, were acquired. A nomogram for the R-R model was also generated with the assistance of this platform. The model performance and its clinical utility were evaluated by calibration curves together with decision curves. DeLong test was performed on the ROC curves of the three models with the utility of MedCalc software (Version 19.0.2) to compare the differences in performance among the models, with a *p*-value less than 0.05 regarded as statistically significant.

## Results

### Clinical and pathological data

Clinical and pathological characteristics of the enrolled subjects are outlined in **Table 2**. Significant differences were observed in terms of age, gender, smoking history, TNM stage, Ki-67 expression, lymph node or pleural metastasis, and STAS between the HGP group and the n-HGP group (*p* = 0.020, *p* = 0.025, *p* = 0.027, *p* < 0.001, *p* < 0.001, *p* < 0.001, *p* < 0.001), yet no remarkable difference could be detected in tumor location and EGFR expression (*p* = 0.360, *p* = 0.586).

**Table 2:** Clinical and pathological characteristics.

Variable	Total (n = 37)	HGP (n = 81)	n- HGP (n = 293)	p
Age(y)				0.020^#^
≤50	103	14	89	
>50	271	67	204	
Sex				0.025^#^
Male	123	35	88	
Female	251	46	205	
Smoking history				0.027^#^
No	345	70	275	
Yes	29	11	18	
Location				0.360^#^
LUL	91	24	67	
LLL	55	15	40	
RUL	126	23	103	
RML	29	7	22	
RLL	73	12	61	
TNM stage				<0.001^*^
I-II	370	77	293	
III-IV	4	4	0	
EGFR+				0.586^#^
No	129	30	99	
Yes	245	51	194	
Ki-67				<0.001^#^
<20%	327	49	278	
≥20%	47	32	15	
Lymph node or pleural metastases				<0.001^*^
No	370	77	293	
Yes	4	4	0	
STAS				
No	370	77	293	<0.001^*^
Yes	4	4	0	

LUL, left upper lobe; LLL, left lower lobe; RUL, right upper lobe; RML, right middle lobe; RLL, right lower lobe; STAS, tumor spread through air spaces; ^#^ Chi-square test; ^*^ Fisher’s exact probability test.

### Construction and verification of a radiological feature-based prediction model

**Figure 3:**
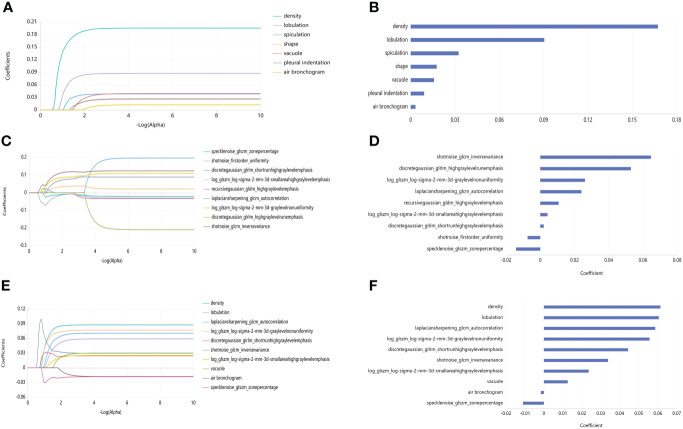
LASSO maps and feature weighted graphs for the three models. **(A)** LASSO map of Radiological model. **(B)** Weighted graph of Radiological model features. **(C)** LASSO map of Radiomics model. **(D)** Weighted graph of Radiomics model features. **(E)** LASSO map of R-R model. **(F)** Weighted graph of R-R model features.

**Figure 4:**
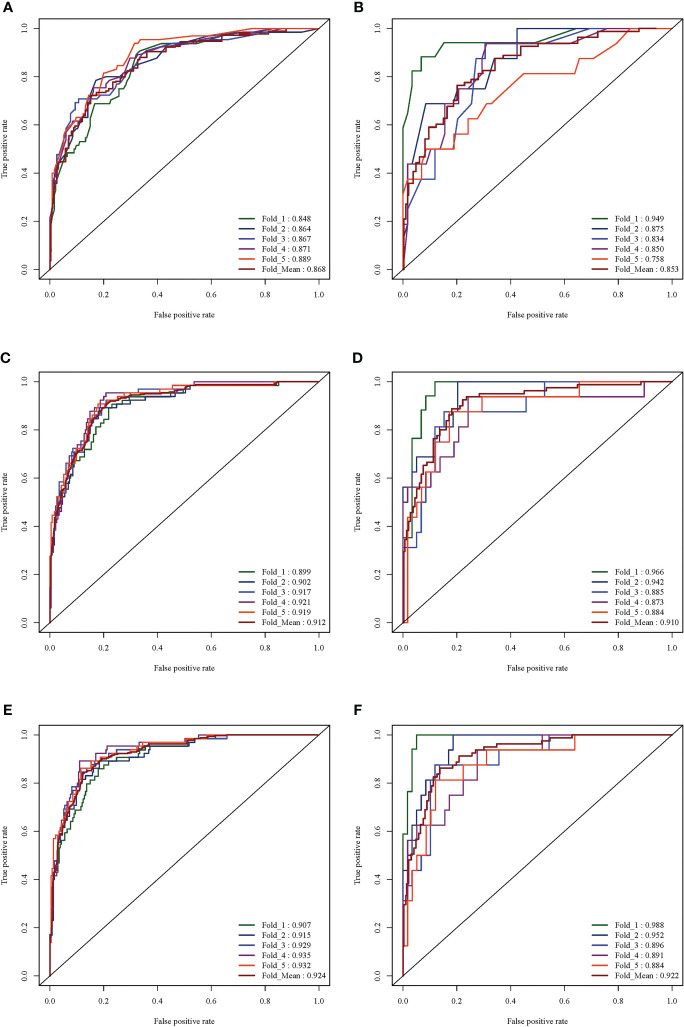
ROC curve analysis results of the three models. **(A)** ROC curve of Radiological model in training set (average AUC = 0.889). **(B)** ROC curve of Radiological mode in validation set (average AUC = 0.758). **(C)** ROC curve of Radiomics mode in training set (average AUC = 0.919). **(D)** ROC curve of the Radiomics model in the validation set (average AUC = 0.884). **(E)** ROC curve of the R-R model in the training set (average AUC = 0.932). **(F)** ROC curve of the R-R model in the validation set (average AUC = 0.88).

Next, 7 radiological features were imported into the platform to generate LASSO maps and weighted graphs (**Figures 3A, B**). A Radiological model was generated with the multi-variable logistic regression method to predict HGP basing on radiological features. This model in the training set showed an AUC value of 0.867 (95%CI: 0.852-0.886), a sensitivity of 77.8%, a specificity of 76.1%, as well as an accuracy of 76.5%; in the validation set, its AUC value was 0.852 (95% CI: 0.811-0.886) and its sensitivity and specificity were 73.8% and 75.1%, respectively, with the accuracy of 74.9%. (**Figures 4A, B**).

### Construction and verification of a radiomics-based prediction model

Additionally, 9 omics features were finally selected by the LASSO method (**Figure 3C**), with the weighted graphs depicted in **Figure 3D**. The Radiomics model was generated with the multi-variable logistic regression method to predict HGP basing on radiomics labels. In the training set, this model had an AUC value of 0.911 (95%CI: 0.897-0.926), together with 85.2% sensitivity, 83.1% specificity, and 83.6% accuracy; additionally, the above-mentioned parameters in the validation set were 0.908 (95% CI: 0.872 - 0.934), 85.1%, 82.2%, and 82.8%, respectively (**Figures 4C, D**).

### Construction and verification of an R-R prediction model

Finally, 10 features were selected by the LASSO method (**Figure 3E**), with the weighted graphs depicted in **Figure 3F**. An R-R model was generated with the multi-variable logistic regression method to predict HGP basing on radiomics features combined with radiological features. In the training set, this model presented an AUC value of 0.923 (95%CI: 0.909 - 0.936), a sensitivity of 87.0%, a specificity of 83.4%, and accuracy of 84.2%; meanwhile, the aforementioned values in the validation set were 0.920 (95% CI: 0.885-0.944), 87.5%, 83.3%, and 84.2%, respectively (**Figures 4E, F**).

### Assessment and clinical application of the R-R prediction model

**Figure 5:**
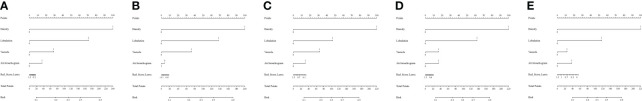
Nomograms of the R-R model. **(A-E)** Fold 1-5 nomograms for the R-R model in the training set. To evaluate the probability of HGP, on each feature axis, a line perpendicular to the point axis was drawn to generate a corresponding point for each feature; the sum of all the points of all features was obtained and then marked on the total score axis, generating a line perpendicular to the risk axis.

**Figure 6:**
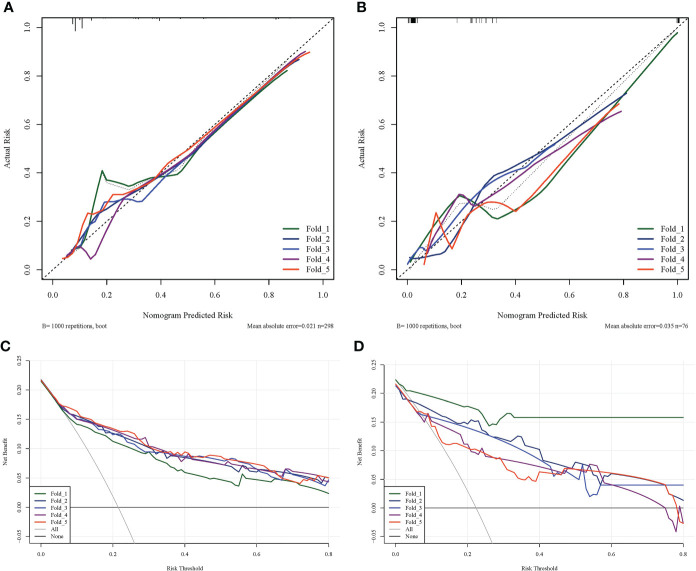
Calibration and decision curves of the R-R model. **(A)** The calibration curve of the R-R model in the training set. **(B)** The calibration curve of the R-R model in the validation set. The fitness of the predicted probabilities of the R-R model to the actual results of the HGP was assessed. The x-axis represents the probability of HGP calculated using the R-R model, while the y-axis represents the actual probability of HGP. The diagonal line represents ideal estimates of the ideal model. **(C)** The decision curve of the R-R model in the training set. **(D)** The decision curve of the R-R model in the validation set. The x-axis represents the threshold probability and the y-axis represents net income.

The prediction performance comparison of the three models is shown in **Table 3**. The results of the Delong test (**Table 4**) revealed that the R-R model was superior to the Radiological model and the Radiomics model in both the training and validation sets. Furthermore, the Radiomics model was superior to the Radiological model. Then, nomograms were developed based on the R-R model (**Figure 5**). The calibration curves for the probability of HGP illustrated the good agreement between nomogram prediction and actual observation (**Figures 6A, B**), indicative of a good calibration performance. The decision curves demonstrated the favorable clinical utility of the R-R model (**Figures 6C, D**).

**Table 3:** Predictive performance of Radiological, Radiomics and R-R model.

	AUC	Sensitivity	Specificity	Accuracy
Development				
Radiological model	0.867	77.8%	76.1%	76.5%
Radiomics model	0.911	85.3%	83.1%	83.6%
R-R model	0.923	87.0%	83.4%	84.2%
Validation				
Radiological model	0.852	73.8%	75.1%	74.9%
Radiomics model	0.908	85.1%	82.2%	82.8%
R-R model	0.920	87.5%	83.3%	84.2%

**Table 4:** Delong test results of Radiological model, Radiomics model and R-R model.

	Z	SE	95%CI	p
Development				
R-R model VS Radiological model	6.029	0.00891	0.0362 - 0.0711	P < 0.0001
R-R model VS Radiomics model	4.509	0.00252	0.00641 - 0.0163	P < 0.0001
Radiomics model VS Radiological model	4.075	0.0104	0.0220 - 0.0627	P < 0.0001
Validation				
R-R model VS Radiological model	3.415	0.0194	0.0283 - 0.104	P = 0.0006
R-R model VS Radiomics model	2.162	0.0054	0.00109 - 0.0223	P = 0.0307
Radiomics model VS Radiological model	2.416	0.0226	0.0103 - 0.0991	P = 0.0157

## Discussion

In this study, we constructed an R-R model on the strength of radiological features integrated with radiomics features to predict the HGP of LUAD, which demonstrated an excellent predictive performance superior to previously reported omics-only models (18, 19). The AUC value of our R-R model in the training set was 0.923, corresponding to a sensitivity of 87.0%, a specificity of 83.4%, and an accuracy of 84.2%. While in the validation set, the model also presented relatively good sensitivity (87.5%) and specificity (83.3%) with an AUC value of 0.920, demonstrating an accuracy of 84.2%. Furthermore, the Decision Curve Analysis (DCA) substantiated the clinical utility of the R-R model. Therefore, this model contributes to better clinical assessment of patient prognosis and the development of accurate clinical decisions.

There have been many reports on the prediction of LUAD aggressiveness based on radiomics, showing a good accuracy (22–24), yet fewer studies focus on the radiomics-based prediction of advanced histological patterns (micropapillary or solid) for LUAD. Ding H et al. have developed a model to predict the micropapillary structure of LUAD (25); there exists, however, a limitation that only micropapillary patterns are predicted, rather than solid patterns. Wang et al. have constructed a radiomics-based model to prognosticate the existence of HGP in early-stage LUAD patients with ground-glass opacity (GGO) (18). However, their study only focused on the lesions with GGO. Our study included GGO and solid nodules, which is more aligned with the actual clinical conditions. Yeonu Choi and He et al. have developed a radiomics model to predict the micropapillary and solid components of LUAD but only radiomics signatures are contained in this model (19, 20). This study is the first report, as far as we know, that HGP can be predicted by a model basing on radiological features integrated with radiomics features in a relatively large dataset. In this study, we constructed an R-R model, which presented higher AUC values than the Radiological and Radiomics models in either the training set or the validation set.

The R-R model involved four radiological features, density, lobulation, vacuolar sign, and air bronchogram. A study has shown that the presence of solid nodules represents an independent prognostic factor (OR = 1.74; 95% CI = 1.10-2.79; *p* = 0.019) in early-stage LUAD patients (26). The formation of the lobulation sign may be caused by uneven cell growth surrounding the tumor or uneven cell differentiation at the tumor margin. Early-stage invasive LUADs without lepidic growth are more prone to lobulation signs (27). The pathological basis of the vacuolar sign is unoccluded small bronchi or alveoli, which may be caused by tumor cells with lepidic growth, and some alveoli and bronchioles are not filled by tumor tissues. An existing study has exhibited a statistical difference in the vacuolar sign among the lepidic, acinar, and papillary adenocarcinomas (*p* = 0.032) (28). In the prediction model of this study, air bronchus sign was negatively correlated with the presence of HGP. Another study has demonstrated a better prognosis of LUAD with air bronchus signs (29). The air bronchus sign of early-stage LUAD is related to the shrinkage and traction owing to intratumoral fibrosis, and the progression of the tumor will compress or infiltrate the bronchus, resulting in the disappearance of the intratumoral bronchi (30). Hence, the negative correlation between HGP and bronchogram may be attributable to the advanced tumors in the HGP group relative to tumors in the n-HGP group, offering an explanation for all 4 stage III-IV adenocarcinomas in the enrolled cases in the HGP group. In light of this finding, we speculated that the tumors gradually developed an HGP during the growth process, which requires verification in more pathological studies.

The three radiological features that were not selected in the R-R model were shape, spiculation and pleural indentation. The formation of the irregular shape of the lesion is related to the inconsistent growth rate of the cells around the tumor, which is also related to the pathological basis of the formation of the lesion lobulation, so we think that the shape of lesions in the R-R model was excluded as a confounding factor in the multivariate logistic regression. The pathological basis of the formation of the spiculation is the proliferation of tumor cells in all directions of the lung parenchyma or the proliferation of the surrounding pulmonary fibrous connective tissue caused by the tumor. The pathological basis of the pleural depression sign is the contraction and traction of the fibrous scar in the tumor. The traction force of the tumor is caused by the reactive fibrosis and scar formation in the tumor. The contraction force is transmitted to the free visceral pleura through the fibrous scaffold structure of the lung. The pleural indentation is related to the location of the lesions. Generally, lesions close to the pleura are more likely to form pleural indentations. Therefore, both the spiculation and the pleural indentation are closely related to the contractile force of fibroblasts (CAF) promoted by tumor cells. Our results also reflect that the growth pattern of the tumor has nothing to do with the contractility of the lesions.

The R-R model contained 6 radiomics features, comprising autocorrelation, gray-level-nonuniformity (GLN), short-run-high-gray-level-emphasis (SRHGLE), inverse-variance (IV), small-area-high-gray-level-emphasis (SAHGLE), and zone-percentage (ZP). Autocorrelation is a measure of the magnitude of the fineness and coarseness of texture. GLN measures the variability of gray-level intensity values in the image. SRHGLE measures the joint distribution of shorter run lengths with higher gray-level values. IV reflects the size of the local change of the image texture. SAHGLE measures the proportion in the image of the joint distribution of smaller size zones with higher gray-level values. ZP measures the coarseness of the texture by taking the ratio of number of zones and number of voxels in the ROI. These radiomics features are all linked to texture heterogeneity, which may correspond to tumor heterogeneity. The lesions in the HGP group presented higher tumor heterogeneity.

These radiomics features are also enrolled in the omics models in many studies. Giovanella et al. have illustrated autocorrelation to be one of two independent predictors of malignant thyroid nodules in a radiomics model basing on FDG PET/CT images (31). Another study has pointed out that GLN is one of the foremost features in omics models for the prediction of high-grade gastric neuroendocrine tumors (32). Zhang et al. have indicated the relevance of SRHGLE to meningioma aggressiveness (33). The article of Wormald et al. has expounded that a radiomics model composed of features such as IV can predict postoperative recurrence of small-volume cervical cancer with an AUC value of 0.808 (34). Chaddad et al. have also stated that inverse-variance is correlated with the survival time of glioblastoma patients (35). In another study, Li has reported SAHGLE as an independent feature to differentiate between primary ovarian granulosa cell tumor and ovarian thecoma-fibrothecoma (OR = 1.034) (36). Weng et al. have elucidated that ZP is one of 4 radiomics features, which can differentiate the aggressiveness of solitary pulmonary nodules that are characterized by part-solid nodules (37).

Our study also has some unexpected clinical and pathological findings, such as the finding that male patients with a history of smoking are more likely to develop HGP, which is in keeping with the latest findings (38). The higher risk of male patients developing an HGP may be relevant to the higher prevalence of smoking in male patients. In this study, 4 patients (presenting TNM stage III-IV tumors) in the HGP group developed lymph node or pleural metastasis. It has been demonstrated that the adenocarcinoma subtype affects lymph node metastasis (LNM) in small-sized lung cancers and that patients with solid histological subtypes less than 1 cm are more likely to develop LNM (39). Additionally, there were 4 cases of STAS in the HGP group in this study, which indicated that LUADs with HGP were more prone to STAS. A recent study has proposed STAS to be a predictor of occult LNM in clinical stage IA LUAD, which is conducive to preoperative selection of the surgical types and of great significance to the improvement of patient prognosis and determination of the surgical methods (40). This study exhibited a higher level of Ki-67 in the HGP group versus the n-HGP group (*p* < 0.001). Ki-67 antigen, a nuclear-associated antigen pertaining to cell proliferation, can reflect the proliferative ability of cells. A higher Ki-67 level correlates with the stronger proliferative potential of tumor cells and further unfavorable patient prognosis (41). Male patients with early-stage LUAD that are manifested as solid nodules show poor prognoses, higher KI-67 expression, and poor differentiation (42), which is concordant with the results of this study. No significant differences were detectable in tumor location and EGFR expression between the two groups in this study.

Several limitations remain in this study. First, there may be inevitable selection bias since this study is a retrospective study. Also, the uneven samples may have a certain impact on the modeling results. Additionally, external validation is not performed for our model. In the future, we will try to construct models with an expanded sample size in a multi-center setting and conduct external validation to increase the generalization ability and robustness of the model.

Taken together, preoperative HGP prediction for LUAD will benefit clinical assessment of patient prognosis and accurate clinical decision-making. The R-R model on the ground of CT radiological features combined with radiomics features exerts excellent diagnostic performance in the prediction of HGP in LUAD less than 3cm in diameter. This potentially offers a reference for clinical diagnosis and surgical therapeutic regimens and aids in improving the prognosis of patients.

## Data availability statement

The original contributions presented in the study are included in the article/supplementary material. Further inquiries can be directed to the corresponding authors.

## Ethics statement

This study was approved by the Ethics Committee of Zhongshan Hospital Affiliated to Fudan University. Written informed consent from the patients/participants OR patients/participants legal guardian/next of kin was not required to participate in this study in accordance with the national legislation and the institutional requirements.

## Author contributions

HD and XY contributed to conception and design. HD organized the database, performed the statistical analysis, and wrote the first draft of the manuscript. HD, LY, and QW contributed to the collection and arrangement of data. LC, XP, and YL contributed to data analysis. XY, LY, LC wrote sections of the manuscript, and MZ contributed to revision and improvement of the manuscript.

## Funding

This work was supported by Major Research Plan of the National Natural Science Foundation of China (Grant No.92059206), National Science Foundation of China (82071990), National Science Foundation of China (81571629), National Science Foundation of China(81301218), Project of Shanghai Science and Technology Commission (19411965200), Policy Guidance Project of Major Science and Technology Plan for Social Development of Xiaoshan District (No. 2021309).

## Conflict of interest

Authors LC, XP, and YL were employed by Shanghai United Imaging Intelligence Co., Ltd., Shanghai, China.

The remaining authors declare that the research was conducted in the absence of any commercial or financial relationships that could be construed as a potential conflict of interest.

## Publisher’s note

All claims expressed in this article are solely those of the authors and do not necessarily represent those of their affiliated organizations, or those of the publisher, the editors and the reviewers. Any product that may be evaluated in this article, or claim that may be made by its manufacturer, is not guaranteed or endorsed by the publisher.
